# Sensor-Derived Parameters from Standardized Walking Tasks Can Support the Identification of Patients with Parkinson’s Disease at Risk of Gait Deterioration

**DOI:** 10.3390/bioengineering13020130

**Published:** 2026-01-23

**Authors:** Francesca Boschi, Stefano Sapienza, Alzhraa A. Ibrahim, Magdalena Sonner, Juergen Winkler, Bjoern Eskofier, Heiko Gaßner, Jochen Klucken

**Affiliations:** 1 Luxembourg Centre for Systems Biomedicine (LCSB), University of Luxembourg, 4367 Esch-sur-Alzette, Luxembourg; 2 Department of Molecular Neurology, University Hospital Erlangen, Friedrich-Alexander-Universität Erlangen-Nürnberg (FAU), 91054 Erlangen, Germany; 3 Fraunhofer Institute for Integrated Circuits IIS, 91058 Erlangen, Germany; 4 Machine Learning and Data Analytics Lab, Department Artificial Intelligence in Biomedical Engineering (AIBE), Friedrich-Alexander-Universität Erlangen-Nürnberg (FAU), 91052 Erlangen, Germany; 5 Institute of AI in Medicine, LMU Klinikum, 85152 Munich, Germany; 6 Translational Digital Health Group, Institute of AI for Health, Helmholtz Zentrum München—German Research Center for Environmental Health, 85764 Neuherberg, Germany; 7 Digital Health and Analytics, Fraunhofer Institute for Integrated Circuits IIS, 91058 Erlangen, Germany; 8 Centre Hospitalier de Luxembourg (CHL), 1210 Luxembourg, Luxembourg

**Keywords:** gait disorders, wearable sensors, machine learning, clinical utility, instrumented gait assessments

## Abstract

**Background:** People with Parkinson’s disease suffer from gait impairments. Clinical scales provide a limited and rater-dependent assessment of gait. Wearable sensors allow an objective characterization by capturing rhythm, pace, and signature patterns. This study investigated if sensor-derived gait parameters have prognostic value for short-term progression of gait impairments. **Methods:** A total of 111 longitudinal visit pairs were analyzed, where participants underwent clinical evaluation and a 4 × 10 m walking test instrumented with wearable sensors. Changes in the UPDRSIII gait score between baseline and follow-up were used to classify participants as Improvers, Stables, or Deteriorators. Baseline group differences were assessed statistically. Machine-learning classifiers were trained to predict group membership using clinical variables alone, sensor-derived gait features alone, or a combination of both. **Results:** Significant between-group differences emerged. In participants with UPDRSIII gait score = 1, Improvers showed higher median gait velocity (0.81 m/s) and stride length (0.80 m) than Stables (0.68 m/s; 0.70 m) and Deteriorators (0.59 m/s; 0.68 m), along with lower stance time variability (3.10% vs. 4.49% and 3.75%; all p<0.05). The combined sensor-based and clinical model showed the best performance (AUC 0.82). **Conclusions:** Integrating sensor-derived gait parameters with clinical score can support the identification of patients at risk of gait deterioration in the near future.

## 1. Introduction

Gait impairments are among the most common and disabling motor deficits experienced by people with Parkinson’s disease (PwP), significantly reducing their quality of life [1]. These gait impairments are mainly caused by dopaminergic loss in Parkinson’s disease (PD), leading to bradykinesia, characterized by slowness of movement and reduced step length, and rigidity. Together, these motor deficits contribute to postural instability and result in typical gait disturbances such as shuffling gait and freezing of gait [2]. Although it is currently not possible to halt or reverse the disease, studies suggest that timely implementation of personalized treatments, pharmacological or non-pharmacological, can benefit PwP by improving balance, gait velocity, and walking endurance [2,3,4].

To achieve this, a precise understanding and detailed characterization of each patient’s gait impairments are needed [5], not only to design appropriate and personalized interventions, but also to identify early the individual risk of short-term deterioration and to evaluate whether the intervention effectively improves gait performance.

Unfortunately, such characterization cannot be fully achieved using the Unified Parkinson’s Disease Rating Scale Section III (UPDRSIII) [6], which assesses gait impairments through a single item scored from 0 to 4 (ranging from no impairment to severe gait dysfunction). This approach lacks granularity and sensitivity and is further limited by the non-linearity of symptom assessment [7,8]. Despite these limitations, the UPDRSIII scale remains the current gold-standard clinical tool for assessing motor symptoms in clinical research.

However, standardized walking tests already used in clinical assessments can be enhanced through the integration of wearable devices equipped with Inertial Measurement Unit (IMU) sensors, typically positioned on the feet, ankles, or trunk [9,10]. Wearables sensors demonstrate strong potential for providing objective, quantifiable, and multiparametric characterization of Parkinsonian gait, capturing aspects such as speed, distance, duration, and variability, while also detecting subtle alterations. Moreover, when combined with artificial intelligence and machine-learning (ML) techniques, these diverse data types have proven valuable in PD research, enabling the identification of hidden feature patterns and supporting a more comprehensive, data-driven understanding of a patient’s clinical status [11,12,13]. Integrating these wearable sensor-derived gait parameters with ML and data-driven analytical methods can support the development of decision-support systems that assist clinicians in delivering timely and individualized interventions, thereby enhancing the clinical applicability of sensor-derived gait parameters in the management of PD [14]. To advance current knowledge, the present retrospective analysis aims to assess the prognostic value of clinical and sensor-derived gait parameters in characterizing gait impairments among PwP who exhibited different trajectories of gait impairment progression. Statistical analyses and machine-learning techniques were employed to test whether these parameters, either individually or in combination, could distinguish individuals whose gait impairments improved, remained stable, or worsened over a short-term observation period.

## 2. Material and Methods

### 2.1. The Dataset

**Figure 1:**
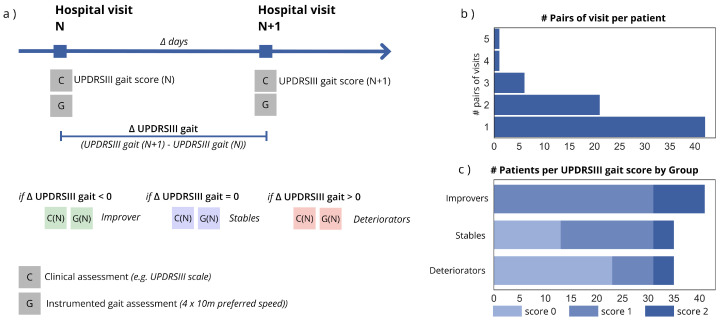
Data collection and dataset description. (**a**) The hospital visit consists of a clinical assessment in which the physician records patient information such as age, gender, and LEDD, fills out the UPDRS scale, and assesses the H&Y score. During the visit, the patient also performs an instrumented gait assessment (4 × 10 m at preferred speed). Changes in the UPDRSIII gait item between consecutive visits are used to assign the baseline data to a specific group. (**b**) Bar plot displaying the distribution of baseline-follow up visits pairs per patient in the study. (**c**) Stacked bar plot displaying the number of Improvers, Stables, and Deteriorators based on their baseline UPDRSIII gait scores at baseline. UPDRS: Unified Parkinson’s Disease Rating Scale Section, C: Clinical scores, G: Gait assessment.

This study analyzed data from longitudinal visits from PwP that were collected between May 2010 and June 2020 in the Department of Molecular Neurology of the University Hospital Erlangen in Germany and was approved by the local ethics committee (Medical Faculty, Friedrich-Alexander University Erlangen-Nürnberg, IRB # 4208, 166_18 B). All patients were diagnosed with a PD diagnosis accordingly to the guidelines of the German Society for Neurology and signed a written informed consent prior to the study. Inclusion criteria comprised Hoehn and Yahr (H&Y) stage < 4, the ability to walk independently (UPDRS gait item < 3), and assessment in the ON medication state, with motor examination using UPDRS part III performed within 30 min prior to gait assessment to minimize the influence of motor fluctuations. Patients with non–Parkinson-related gait impairments, atypical Parkinsonian syndromes, major neurological comorbidities, or severe cognitive impairment were excluded [10]. We analyzed pairs of consecutive clinical visits [10], with the earlier visit defined as the baseline and the subsequent visit as the follow-up. Eligible visit pairs were required to have an interval of 30 to 365 days. Given the primary objective of assessing the prognostic value of clinical and sensor-derived gait parameters in relation to short-term changes in gait performance, each visit pair was treated as an independent observation to examine baseline characteristics associated with subsequent trajectories of gait impairment progression. Each visit consisted of a clinical assessment, which included the patient’s age at the time of the study, disease duration, Hoehn and Yahr (H&Y) stage, gender, date of the visit, levodopa equivalent daily dose (LEDD), and the UPDRSIII items. For this analysis, we specifically retained the UPDRSIII total score along with the UPDRSIII gait, bradykinesia, postural stability, and arise-from-chair items. The selection of UPDRSIII items was based on their relevance to postural instability and gait dysfunction [15,16]. During each visit, PwP performed a 4 × 10 m walking test. Patients were instructed to walk at their preferred speed while wearing IMU sensors on their shoes. Data were recorded using Shimmer sensors [10] and the Mobile GaitLab system (Portabiles HealthCare Technologies GmbH, Erlangen, Germany) [17]. Gait parameters were extracted using the gaitmap Python 3.11.14 package [18]. The following gait features were computed and averaged across the 4 × 10 m test: gait velocity (m/s), swing time (s), stance time (s), stride length (m), initial contact (IC) angle (°), terminal contact (TC) angle (°), maximum lateral excursion (m), and maximum sensor lift (m). To assess gait variability, the coefficient of variation (CV) was calculated for gait velocity, swing time, stance time, and stride length for each recording. Gait velocity, gait velocity CV, stride length, stride length CV, maximum lateral excursion, and maximum sensor lift were normalized to the patient’s height. PwP were assessed in the ON medication state. The UPDRSIII gait score, which has low assessment error and-inter operator variability [19] and whose change can be considered clinically meaningful [20], was used to identify three trajectory groups of gait progression: Improvers, Stables, and Deteriorators. According to the longitudinal change in this item between the baseline and follow-up visits, each data collected at a baseline visit was assigned to one of the three groups based on the direction of change in the ΔUPDRSIII gait score between consecutive visits, corresponding to negative, neutral, or positive changes, respectively, Figure 1. This grouping was also justified, as no significant statistical changes in the LEDD were observed between the baseline and follow-up visits across the groups.

### 2.2. Statistical Analysis

A primary statistical analysis was conducted on the clinical and gait parameters of the overall dataset, while a secondary analysis was performed on three subsets obtained by stratifying participants according to their UPDRSIII gait scores at baseline. The aim was to assess significant differences at baseline in clinical and gait parameters among the three gait progression trajectory groups. For comparison across the three groups, a one-way ANOVA was performed when the data met the assumptions of normality and homogeneity of variance; otherwise, a Kruskal–Wallis test was used. The effect size was calculated for comparisons with a *p*-value < 0.05 using eta squared ($\eta^{2}$). Post hoc analyses were conducted to explore pairwise group differences: Tukey’s HSD test was applied following ANOVA, whereas Dunn’s test was used after Kruskal–Wallis tests. *p*-values from post hoc analyses were corrected for multiple comparisons using the Benjamini–Hochberg method [21]. For comparison across two groups, an independent *t*-test was performed if the data met the assumptions of normality and homogeneity of variance; otherwise, a Mann–Whitney U test was used. The effect size was calculated for comparisons with a *p*-value < 0.05 using Cohen’s d after the independent *t*-test or using r after the Mann–Whitney U test. *p*-values were then corrected using the Benjamini–Hochberg correction. The following Python packages were used: scipy.stats for statistical functions and tests such as the Shapiro–Wilk test and *t*-test; statsmodels and scikit.posthocs for advanced statistical models such as post hoc tests and multiple comparison correction.

### 2.3. Machine-Learning Models

We evaluated the performance of three Random Forest (RF) models trained to identify the gait progression trajectory groups. The first model included only clinical features, the second included only gait features, and the third combined both clinical and sensor-derived gait features. The final goal was to characterize the trajectory groups by exploring feature interactions and their relative contribution to group discrimination. Prediction targets were based on the classification of participants as Improvers, Stables, or Deteriorators, as defined in Figure 1 and previously used in the statistical analysis. UPDRSIII gait item was included in the pool of clinical features. Although this information introduces some constraints which facilitate the task of ML model (such as individuals with UPDRS gait score 0 at baseline cannot deteriorate), it represents a key piece of clinical information the physician has available for the prognosis during the baseline visit. We selected the RF as the ML model in our study, since it showed the best performance in terms of Area Under the Curve (AUC) in predicting UPDRSIII score [12], PD stages [22,23] and specific motor symptoms using sensor derived gait features [24]. Although the data derive from a longitudinal cohort, the machine-learning analysis was conducted on baseline–follow-up visit pairs defined over a fixed time interval, each treated as an independent short-term gait progression trajectory. This pair-based formulation avoids overlapping temporal windows and does not aim to model subject-specific longitudinal trajectories across multiple time points. Under this study design, Random Forest classifiers assuming independent samples are appropriate and have been shown to be suitable for longitudinal biomedical data reformulated as independent observation units, in line with recent methodological recommendations [25]. Each classifier was trained and tested on a total of 72 hyperparameter combinations [23]. Hyperparameter combinations spanned the number of trees (10, 20, 50, 150, 200), minimum leaf size (1, 2, 5) [26], and the number of features. Feature selection was performed by testing fixed sizes of 4, 6, 8, 10, and 12 features, representing approximately half or less of the available subsets. For smaller subsets (only clinical features), the upper limit matched the subset size when below this range. The models were trained with labels corresponding to the group membership of the samples, using a cost matrix that assigned double the cost for misclassifying Improvers as Deteriorators (and vice versa) compared to misclassifying Improvers as Stables or Deteriorators as Stables. Feature selection was performed using the ReliefF [27], which works effectively with multi-class problems and can capture complex, non-linear relationships among features. It has also already been applied in various studies related to longitudinal changes in Parkinson’s disease [28]. For each hyperparameter combination, a 10-Fold cross-validation was performed for 10 iterations to obtain evaluation metrics such as AUC scores and accuracies. To ensure that each fold preserved the distribution of both outcome classes and baseline gait severity, stratified cross-validation was performed using a combined stratification scheme based on class labels and UPDRSIII gait scores. The hyperparameter combination with the highest average AUC score was selected as the best for both models. Significant differences in the model performance metrics across the two datasets were assessed using either a paired *t*-test or a Wilcoxon signed-rank test, depending on whether the assumption of normality was satisfied. Normality of the paired AUC differences was evaluated using the Shapiro–Wilk test. If the data met normality assumptions, a paired *t*-test was applied; otherwise, the non-parametric Wilcoxon signed-rank test was used. All statistical tests were two-sided, and significance was determined at an α level of 0.05. The ML models were developed in Python, using skrebate library for the ReliefF algorithm and the sklearn package for stratified K-Fold model selection, as well as for implementing the Random Forest Classifier and AUC scores.

## 3. Results

A total of 111 valid visit pairs from 71 PwP were analyzed, comprising 41 Improvers, 35 Stables, and 35 Deteriorators. Demographics and clinical data for the overall population are presented in Table 1.

### 3.1. Significant Differences in Clinical and Sensor Derived Gait Parameters at Baseline

Analyzing the clinical parameters at baseline, we found that the UPDRSIII gait and bradykinesia scores are statistically different, after multiple comparison correction, between Improvers and Stables, as well as between Improvers and Deteriorators. Importantly, neither the time interval between visits nor the disease stage differed significantly among the groups, allowing us to exclude these variables as potential confounders influencing the observed gait impairment trajectories. While the statistical difference observed in the UPDRSIII gait score between Improvers and the other two groups might be biased by the fact that individuals categorized as Improvers, by definition, cannot present a baseline gait score of zero, the statistical difference in bradykinesia suggests that this cardinal motor symptom should be considered when evaluating the progression of gait impairments. No statistically significant differences were found between Stables and Deteriorators, Table 2.

Gait parameters at baseline were then analyzed. Although no statistical differences were found between Stables and Deteriorators, as between Improvers and Deteriorators, all variability sensors derived gait parameters were found statistically different between Improvers and Stables, Table 3.

Improvers exhibited significantly lower variability in gait parameters compared to Stables, along with generally higher median values for gait velocity, stride length, and IC angle relative to both Stables and Deteriorators. Despite having worse baseline clinical characteristics in terms of bradykinesia, Improvers demonstrated more favorable sensor-derived gait parameters than Stables. It is important to mention that the lack of statistically significant differences between Improvers and Deteriorators may be attributed to a confounding effect introduced by the initial level of gait impairment severity. This confounding effect could mask underlying differences in gait parameters, as gait impairments in this analysis can range from absent to mild (score 0 to 2 in UPDRSIII gait item). To account for this confounding effect, we used the categorical nature of the UPDRSIII gait score at baseline to analyze clinical and sensor derived gait parameters in patients with the same baseline score. This generated three sub-datasets. The first, consisting of PwP with gait score of 0 at baseline composed of 13 Stables and 23 Deteriorators. The second with participants with gait score of 1, accounted for 31 Improvers, 18 Stables, and 8 Deteriorators. Finally, the most severe group presented a gait score of 2 at baseline, with 10 Improvers, 4 Stables, and 4 Deteriorators. While no statistically significant differences in clinical parameters were observed between the groups Table 4, the analysis revealed new insights from the gait parameters, Table 5.

For patients with a UPDRSIII gait score of 1, gait velocity, stance time, stride length, and stance time CV were statistically different between Improvers and Stables, as well as between Improvers and Deteriorators, after multiple comparison correction. Improvers showed higher values in gait velocity, stride length, and reduced stance time and stance time variability compared to both Stables and Deteriorators. Furthermore, the TC angle and the remaining gait variability features were statistically different between Improvers and Stables, with Improvers showing higher absolute values for TC angles and reduced variability compared to Stables. Although not statistically significant differences after multiple comparison correction, the lowest *p*-values and largest effect sizes were observed in gait velocity, stride length, and TC angle between Stables and Deteriorators with UPDRSIII gait score of 0 at baseline and between Improvers and Deteriorators with UPDRSIII gait score of 2 at baseline. The patterns observed in these patients were similar to that seen in patients with a UPDRSIII gait score of 1 at baseline, where reduced gait velocity, stride length, and absolute TC angle were also noted in PwP that did not show an improvement or stability in gait impairments.

**Figure 2:**
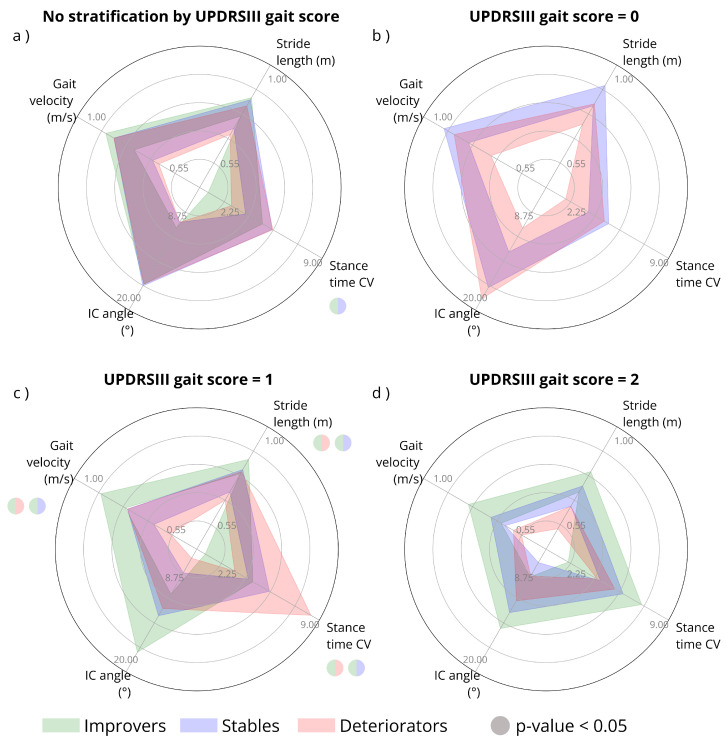
Spider plots illustrating the distribution of four gait parameters (stride length, gait velocity, IC angle, and stance time CV) based on the 25th and 75th percentiles. (**a**) Non-stratified dataset; (**b**) stratified by UPDRSIII gait score 0; (**c**) stratified by UPDRSIII gait score 1; (**d**) stratified by UPDRSIII gait score 2. Percentile ranges for Improvers, Stables, and Deteriorators indicated in green, blue, and red, respectively. Radial axes for stride length and gait velocity range from 0.4 to 1 m; for IC angle from 5° to 20°; for stance time CV from 0 to 9. Dots indicate significant differences between two groups, with the specific groups color-coded inside the circle. IC: initial contact, CV: coefficient of variation.

The spider plots in Figure 2 illustrate, as example, the 25th–75th percentile distributions of four gait parameters, gait velocity, stride length, IC angle, and stance time CV, for the full dataset and after the stratification by UPDRS gait score at baseline.

It is possible to observe in Figure 2a that without stratification by UPDRSIII gait score at baseline there is an overlap in the distribution of Improvers, Stables, and Deteriorators. This overlap tends to decrease when considering the UPDRSIII gait score at baseline, as shown in Figure 2b–d.

### 3.2. ML Performances in Identifying the Trajectory Groups of Gait Progression

**Figure 3:**
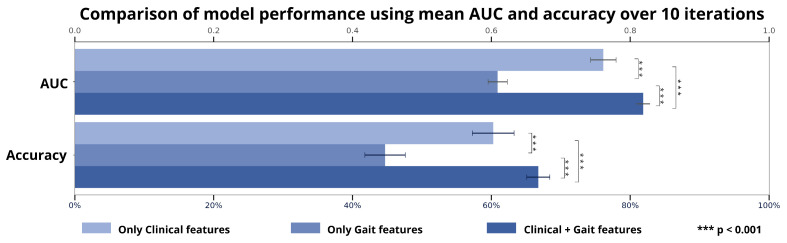
Overview of ML model performances. Bar plots show the average AUC and accuracy for all three classifiers over 10 iterations, with *** indicating *p* < 0.001. AUC: area under the curve.

The best predictive performance in terms of AUC (0.82 ± 0.01) and accuracy (67.21 ± 1.92) was achieved by the RF model combining both clinical and gait parameters, with optimal hyperparameters: 8 features, 200 trees, and a minimum leaf size of 1. This was followed by the model using only clinical parameters (AUC = 0.78 ± 0.01; accuracy = 60.72 ± 3.07; hyperparameters: 10 features, 150 trees, minimum leaf size of 1) and the model using only sensor-derived gait parameters, which showed the lowest performance (AUC = 0.62 ± 0.01; accuracy = 44.59 ± 1.86; hyperparameters: 8 features, 200 trees, minimum leaf size of 1). The independent *t*-test revealed statistically significant differences in AUC scores (*p* < 0.001) and accuracies (*p* < 0.001) across all three models, Figure 3.

**Figure 4:**
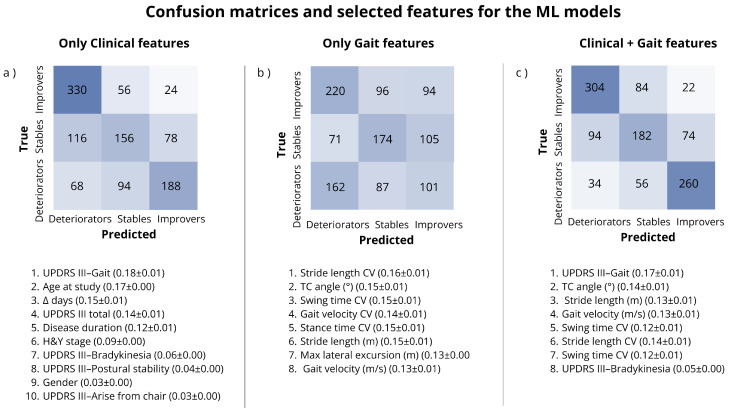
Cumulative confusion matrices of the three ML models and corresponding selected features. (**a**) for only clinical features, (**b**) for only sensor derived gait features, and (**c**) for clinical and gait features. Confusion matrices share the same color scale. AUC: area under the curve, H&Y: Hoehn and Yahr, UPDRS: Unified Parkinson’s Disease Rating Scale, TC: terminal contact, CV: coefficient of variation.

The confusion matrices for the three models, together with the corresponding selected features, are reported in Figure 4.

To assess the potential confounding effect of including the UPDRSIII gait item, we also tested a model in which this item was incorporated into the Postural Instability and Gait Difficulty score (PIGD). The analysis produced similar results, see Appendix A, with the combined model performing slightly better overall, although some misclassification errors increased. For this reason, we decided to retain the UPDRSIII gait score at baseline as an individual input feature.

## 4. Discussion

This study leveraged statistical and ML models to investigate whether patterns captured through sensor-derived gait parameters can be informative about different trajectories of gait impairment progression in PwP. Our results suggest that these features possess a certain degree of prognostic value. However, to maximize the impact of these digital biomarkers, it is critical to interpret them in conjunction with clinical information. In fact, the statistical analysis showed clearer and more explainable patterns after the stratification of our dataset by UPDRSIII gait score. Furthermore, the best ML predictive performance was achieved by incorporating both clinical and sensor derived gait parameters, whereas the model trained exclusively on the gait parameters obtained the worst results. Hence, these results support the idea that accurate clinical characterization is essential for interpreting sensor-derived gait parameters, particularly in the context of developing decision-support systems for individualized intervention. Our findings align with previous works in literature. In their paper, Hill et al. [9] suggested that gait impairments characterization in the clinic can be enhanced by sensor-derived gait parameters as a complement to the UPDRSIII. A second study by Godi et al. [29] demonstrated that stratifying patients using the H&Y score revealed gait pattern differences between early- and late-stage PD that would otherwise be missed, reinforcing the value of combining clinical stratification with sensor data. Additionally, the study by Hähnel et al. [11], which focused on identifying Parkinson’s progression rates, also highlighted a group of sensor-derived gait parameters as significantly different between slow and fast progressors, including the same gait features, gait velocity, stride length, TC angle and stance time, that have been identified in this study. In line with these findings, ML-based feature selection in our study showed that, besides the UPDRS-III gait item, stride length, gait velocity, and TC angle were the most relevant sensor-derived gait features for trajectory discrimination. These features capture distinct aspects of gait, including foot progression and foot–ground interaction. While gait velocity is an established key parameter in PD studies [30], stride length and TC angle have also shown promising results for monitoring disease progression and severity under supervised assessment strategies [31,32]. Moreover, TC angle has been reported to be sensitive to treatment effects [32]. One of the key contributions of this manuscript is the generation of reference values for sensor-derived gait parameters, which we found to be significant for distinguishing trajectories of gait impairment progression. Although further research is needed, Baudendistel S. [33] indicates a minimal clinically relevant difference in gait velocity of 8.2 cm/s for standardized gait assessment. Comparable or larger differences were found in our work between the groups of Improvers, Stables and Deteriorators after stratifying the dataset. Such delta in gait velocity is associated with a reduction of stride length, or an increase in stride time, or both simultaneously. A shorter stride length is primarily attributed to a reduced ability to move the body forward effectively, whereas an increased stride time, if driven by an increased stance phase, is associated with postural instability, as prolonging the time both feet remain on the ground can help PwP to maintain their posture [34]. When considering patients with UPDRSIII gait score of 1 at baseline, the distribution in our dataset suggests that patients that will deteriorate present simultaneously a reduced stride length (more than 7.2 cm of difference which is considered clinically relevant [33]) and an increased stance time compared to patients that have shown an improvement in gait impairments. For Deteriorators with UPDRSIII gait score of 2 instead, gait velocity and stride length shown a clinically significant difference between both Stables and Improvers, whereas comparable values of stance time were observed. However, it is important to mention that these results could be influenced by the limited sample size of patient with UPDRSIII gait score of 2 at baseline, composed by only 18 individuals across all groups. Among Deteriorators with baseline UPDRSIII gait scores of 1 or 2, higher values of gait variability parameters compared to Improvers were observed, which are typically associated with gait instability and increased fall risk. Even though Deteriorators with a baseline UPDRSIII gait score of 0 exhibited clinically relevant differences in gait velocity and stride length, as well as comparable stance time to Stable patients with the same score, the Stables group demonstrated increased variability across all gait parameters. Since an UPDRSIII gait score of 0 indicates the absence of observable gait impairments, these findings suggest that PwP who are likely to deteriorate may reduce their gait velocity as a compensatory strategy to enhance postural and balance control. In contrast, Stables may exhibit increased gait variability as an intrinsic manifestation of the disease itself [35], despite not showing gait impairments during the visual clinical assessment. As second key clinical factor for the identification of patients at risk identified by the ML model features selection is the severity of bradykinesia, which is primarily linked with the sensor-derived gait speed, but also with variability features that reflect postural instability [35]. Both our statistical and ML analysis showed how the most challenging group to identify are the Stables, as the statistical analysis did not show any statistically significant separation from the Deteriorators group and resulted in the hardest to predict by the ML model. In fact, the best predictive model, trained on both clinical and gait features, achieved a sensitivity of 74% for both Improvers and Deteriorators, but only 52% for the Stables. Although, for all groups, the model achieved specificity above 80%. Observing the confusion matrices in Figure 4, most of the errors occurred within the Stables group, with fewer misclassifications between Improvers and Deteriorators. In this context, the present findings may be relevant for the future design and evaluation of trial outcomes involving sensor-derived gait parameters. Randomized rehabilitation studies, such as those by [36], used inertial sensors to assess physiotherapy or treadmill training in people with Parkinson’s disease and reported improvements in gait velocity and stride length. Similarly, sensor-derived gait velocity and stride length were key outcome measures in the evaluation of wearable biofeedback devices designed to improve gait patterns in Parkinson’s disease [37], as well as in exploratory observational studies investigating multimodal complex treatment [38]. We believe that the stratification approach proposed in this work could support the extension of similar studies toward individualized treatment plans tailored to patients’ specific needs. Despite the promising results, it is important to recognize the limitations of our observational study. Notably, there is currently no consensus on the optimal time interval required to assess changes in disease progression [39,40,41]. Ideally, all follow-up visits should occur after a standardized time interval, this was not the case for our study. Interestingly, the ML model, for our dataset, did not select the time between baseline and follow-up visits as a relevant feature to distinguish the three groups. A second limitation of this study is that detailed treatment regimens were not available for analysis. Although no statistically significant changes in LEDD were observed across follow-up visits and gait changes have been reported to be largely unrelated to dopaminergic medication adjustments [2,35,42], individual improvements may still have resulted from specific non-pharmacological interventions, while deterioration could have been influenced by unpredictable traumatic events. In addition, several factors known to affect gait in people with Parkinson’s disease were not available in this study. Although age and sex did not differ significantly between groups, gait velocity is known to be affected by aging, and future studies should aim for a balanced representation of male and female participants. Comorbid conditions, including other neurological disorders and metabolic diseases such as diabetes, may further deteriorate gait, as may cognitive decline, which has been associated with worsening gait performance [9,43]. Moreover, family and social support have been shown to enhance motivation and treatment adherence, potentially contributing to reported improvements [44,45]. Sensory function (vision and hearing) and gait assessment under uneven or real-world conditions were not explored, yet are critical elements for future wearable-based trials investigating gait impairments in PwP. Finally, it is important to acknowledge the risk of circularity in the ML analysis, where the UPDRSIII gait score at baseline is used as predictor but also to generate the labels to estimate by the model. This could potentially enhance the absolute performance of the models. However, it is essential to underline how the goal of the ML analysis in this paper was not to achieve optimal prediction but rather to evaluate if sensor-derived gait features possess prognostic power. In this context, the key metric is the delta in performance observed when such parameters are integrated with clinical scores. The fact the comparison is made with the clinical model, which is trained also with the UPDRSIII gait score, should mitigate potential bias.

## 5. Conclusions

Although further validation in larger cohorts is needed, these findings represent an initial step toward the development of decision-support systems that assist healthcare professionals in clinical practice. By complementing standard patient assessments with data-driven insights on gait impairment progression, this approach can enhance clinical decision-making and guide timely, personalized interventions aimed at improving patient quality of life.

## Figures and Tables

**Table 1:** Median and interquartile range (IQR) are presented for the 71 PwP at their first visit in the study.

Feature	Median (IQR)
Age at study (years)	64 (14)
Height (cm)	171 (10)
Weight (kg)	74 (14)
Disease duration (years)	5 (7)
H&Y score	2 (2)
Gender	F: 25; M: 46
Δdays	168 (166)
LEDD (mg/day)	360 (644)
UPDRSIII total score	18 (12)

F: number of females; M: number of males; H&Y: Hoehn and Yahr scale; LEDD: Levodopa Equivalent Daily Dose; UPDRSIII: Unified Parkinson’s Disease Rating Scale part III; Delta days: difference in days between baseline and follow-up visit.

**Table 2:** Median [IQR] values are reported for Improvers, Stables, and Deteriorators.

Feature	Improvers (n = 41)	Stables (n = 35)	Deteriorators (n = 35)	*p*-Value	Effect Size ($\eta^{2}$)	*p* Impr.—Stables	*p* Impr.—Deter.	*p* Stables—Deter.
Age at study (years)	61 (15)	63 (14)	70 (12)	0.031	0.062	1.000	0.110	0.729
Disease duration (years)	5 (7)	6 (8)	6 (8)	0.738	<0.001	–	–	–
H&Y score	2 (1)	2 (1)	2 (1)	0.255	0.007	–	–	–
Gender (F/M)	F: 15; M: 26	F: 9; M: 26	F: 14; M: 21	0.421	<0.001	–	–	–
Δdays	99 (153)	196 (175)	189 (174)	0.145	0.017	–	–	–
LEDD (mg/day)	460 (594)	420 (516)	480 (648)	0.978	<0.001	–	–	–
UPDRSIII total score	18 (13)	15 (8)	14 (8)	0.037	0.042	0.390	0.072	0.981
UPDRSIII gait score	1 (0)	1 (1)	0 (1)	<0.001	0.246	0.017	<0.001	0.237
UPDRSIII bradykinesia	1 (1)	1 (0)	1 (0)	0.002	0.094	0.024	0.024	1.000
UPDRSIII arise from chair	0 (1)	0 (1)	0 (1)	0.895	<0.001	–	–	–
UPDRSIII postural stability	1 (0)	0 (1)	1 (1)	0.045	0.039	0.072	0.615	0.728

Statistical analyses include ANOVA/Kruskal–Wallis tests with effect sizes eta squared ($\eta^{2}$) and post hoc *p*-values (Tukey’s HSD/Dunn’s test). Pairwise *p*-values were corrected using the Benjamini–Hochberg method. Statistical significance was defined as *p*-value <0.05. (F: number of females; M: number of males; N: group numerosity; – Not performed, post hoc tests were not conducted when the overall test was not significant). $\eta^{2}$ values were interpreted according to Cohen’s guidelines as small (∼0.01), medium (∼0.06), and large (≥0.14).

**Table 3:** Median [IQR] values of sensor-derived gait parameters for Improvers, Stables, and Deteriorators.

Feature	Improvers (n = 41)	Stables (n = 35)	Deteriorators (n = 35)	*p*-Value	Effect Size ($\eta^{2}$)	*p* Impr.—Stables	*p* Impr.—Deter.	*p* Stables—Deter.
Gait velocity (m/s)	0.78 (0.14)	0.74 (0.19)	0.72 (0.22)	0.046	0.038	0.330	0.091	0.811
Swing time (s)	0.37 (0.03)	0.38 (0.04)	0.37 (0.04)	0.809	0.004	–	–	–
Stance time (s)	0.63 (0.09)	0.67 (0.05)	0.65 (0.11)	0.139	0.018	–	–	–
Stride length (m)	0.78 (0.09)	0.77 (0.14)	0.75 (0.14)	0.075	0.030	–	–	–
IC angle (°)	14.17 (6.91)	12.82 (7.81)	12.26 (7.59)	0.642	<0.001	–	–	–
TC angle (°)	−66.78 (8.39)	−65.96 (8.63)	−64.10 (10.61)	0.032	0.045	0.350	0.066	0.565
Max lateral excursion (m)	0.03 (0.01)	0.02 (0.01)	0.02 (0.01)	0.336	0.002	–	–	–
Max sensor lift (m)	0.11 (0.02)	0.12 (0.01)	0.11 (0.01)	0.346	0.001	–	–	–
Gait velocity CV (%)	4.07 (5.21)	6.06 (2.00)	5.57 (3.13)	0.002	0.096	0.008	0.330	0.267
Swing time CV (%)	2.94 (3.90)	4.30 (1.37)	3.61 (2.65)	0.004	0.083	0.012	0.306	0.331
Stance time CV (%)	3.10 (3.99)	4.49 (1.96)	3.75 (3.06)	0.012	0.063	0.027	0.429	0.330
Stride length CV (%)	3.23 (3.90)	5.53 (2.30)	4.31 (2.72)	<0.001	0.131	0.002	0.300	0.162

Median [IQR] values are reported for Improvers, Stables, and Deteriorators. Statistical analyses include ANOVA/Kruskal–Wallis tests with effect sizes eta squared ($\eta^{2}$) and post hoc *p*-values (Tukey’s HSD/Dunn’s test). Pairwise *p*-values were corrected using the Benjamini–Hochberg method. Statistical significance was defined as *p*-value < 0.05. (–): Not performed; post hoc tests were not conducted when the overall test was not significant. $\eta^{2}$ values were interpreted according to Cohen’s guidelines as small (∼0.01), medium (∼0.06), and large (≥0.14).

**Table 4:** Median [IQR] values of clinical parameters stratified by UPDRSIII gait score for Improvers, Stables, and Deteriorators.

Feature	UPDRSIII Gait Score	Impr. (n = 41)	Stables (n = 35)	Deter. (n = 35)	*p*-Value	Effect Size ($\eta^{2}$)	*p* Impr.—Stables	*p* Impr.—Deter.	*p* Stables—Deter.
Age at study (years)	0		57 (7)	68 (12)		<0.001			0.342
	1	61 (13)	66 (14)	71 (10)	0.056	0.070	–	–	–
	2	70 (11)	74 (7)	72 (5)	0.380	<0.001	–	–	–
Disease duration (years)	0		7 (4)	4 (6)		0.250			0.342
	1	5 (5)	7 (10)	9 (6)	0.201	0.022	–	–	–
	2	8 (6)	4 (4)	13 (12)	0.434	<0.001	–	–	–
H&Y score	0		1 (1)	2 (1)		0.296			0.342
	1	2 (1)	2 (0)	2 (1)	0.716	<0.001	–	–	–
	2	3 (0)	3 (0)	4 (1)	0.258	0.165	–	–	–
Gender (F/M)	0		F: 7; M: 6	F: 9; M: 14		0.121			0.456
	1	F: 13; M: 18	F: 2; M: 16	F: 3; M: 5	0.079	0.057	–	–	–
	2	F: 2; M: 8	F: 0; M: 4	F: 2; M: 2	0.247	0.053	–	–	–
Δdays	0		210 (175)	189 (145)		0.338			0.422
	1	99 (109)	214 (176)	175 (163)	0.191	0.024	–	–	–
	2	122 (183)	100 (43)	322 (49)	0.093	0.184	–	–	–
LEDD (mg/day)	0		420 (349)	257 (478)		0.184			0.422
	1	400 (479)	415 (548)	793 (386)	0.158	0.031	–	–	–
	2	845 (704)	657 (102)	672 (496)	0.636	0.059	–	–	–
UPDRSIII total score	0		8 (11)	12 (9)		<0.001			0.705
	1	17 (12)	18 (8)	15 (6)	0.687	<0.001	–	–	–
	2	22 (12)	19 (12)	20 (13)	0.816	0.027	–	–	–
UPDRSIII bradykinesia	0		1 (1)	1 (1)		0.060			0.342
	1	1 (1)	1 (0)	1 (0)	0.409	<0.001	–	–	–
	2	2 (1)	1 (1)	2 (0)	0.423	<0.001	–	–	–
UPDRSIII arise from chair	0		0 (0)	0 (0)		0.143			0.422
	1	0 (1)	0 (1)	1 (1)	0.279	0.010	–	–	–
	2	0 (1)	1 (0)	0 (1)	0.213	0.073	–	–	–
UPDRSIII postural stability	0		0 (0)	0 (1)		0.176			0.422
	1	1 (1)	0 (1)	1 (0)	0.252	0.014	–	–	–
	2	1 (1)	2 (1)	2 (1)	0.702	<0.001	–	–	–

Median [IQR] values are reported for Improvers, Stables, and Deteriorators. Statistical analyses include ANOVA/Kruskal–Wallis tests with effect sizes eta squared ($\eta^{2}$) and post hoc *p*-values (Tukey’s HSD/Dunn’s test). Pairwise *p*-values were corrected using the Benjamini–Hochberg method. Statistical significance was defined as *p*-value < 0.05. (–): Not performed; post hoc tests were not conducted when the overall test was not significant. $\eta^{2}$ values were interpreted according to Cohen’s guidelines as small (∼0.01), medium (∼0.06), and large (≥0.14).

**Table 5:** Median [IQR] values of sensor-derived gait parameters stratified by UPDRSIII gait score for Improvers, Stables, and Deteriorators.

Feature	UPDRSIII Gait Score	Impr. (n = 41)	Stables (n = 35)	Deter. (n = 35)	*p*-Value	Effect Size ($\eta^{2}$)	*p* Impr.—Stables	*p* Impr.—Deter.	*p* Stables—Deter.
Gait velocity (m/s)	0		0.82 (0.12)	0.76 (0.18)		0.618			0.209
	1	0.81 (0.13)	0.68 (0.12)	0.59 (0.21)	<0.001	0.281	0.006	0.007	0.751
	2	0.74 (0.16)	0.62 (0.08)	0.54 (0.04)	0.045	0.340	1.000	0.427	1.000
Swing time (s)	0		0.37 (0.04)	0.36 (0.03)		0.333			0.460
	1	0.37 (0.02)	0.38 (0.04)	0.40 (0.03)	0.070	0.062	–	–	0.070
	2	0.38 (0.06)	0.37 (0.02)	0.37 (0.02)	0.174	0.207	–	–	0.175
Stance time (s)	0		0.63 (0.08)	0.63 (0.09)		<0.001			0.875
	1	0.62 (0.08)	0.67 (0.06)	0.73 (0.05)	0.001	0.244	0.028	0.006	0.314
	2	0.66 (0.09)	0.67 (0.01)	0.68 (0.04)	0.972	0.004	–	–	–
Stride length (m)	0		0.87 (0.09)	0.79 (0.10)		0.986			0.091
	1	0.80 (0.08)	0.70 (0.11)	0.68 (0.14)	<0.001	0.251	0.019	0.015	0.911
	2	0.74 (0.09)	0.64 (0.09)	0.55 (0.11)	0.007	0.485	1.000	0.092	0.911
IC angle (°)	0		16.07 (4.37)	13.79 (8.65)		0.173			0.091
	1	14.40 (7.12)	11.25 (5.26)	10.46 (6.14)	0.021	0.106	0.088	0.059	0.751
	2	10.82 (6.32)	9.21 (6.10)	9.05 (3.03)	0.725	<0.001	–	–	–
TC angle (°)	0		–69.93 (3.50)	–64.93 (8.17)		0.387			0.127
	1	–67.79 (9.09)	–63.84 (5.61)	–67.39 (14.24)	0.035	0.088	0.031	0.522	0.690
	2	–65.10 (7.62)	–59.61 (2.20)	–51.27 (8.84)	0.007	0.487	1.000	0.092	0.911
Max lateral excursion (m)	0		0.03 (0.02)	0.02 (0.01)		0.107			0.638
	1	0.03 (0.01)	0.02 (0.01)	0.03 (0.01)	0.408	<0.001	–	–	–
	2	0.03 (0.01)	0.02 (0.01)	0.02 (0.01)	0.057	0.318	–	–	–
Max sensor lift (m)	0		0.12 (0.01)	0.11 (0.01)		0.119			0.800
	1	0.11 (0.01)	0.11 (0.02)	0.12 (0.01)	0.606	<0.001	–	–	–
	2	0.11 (0.04)	0.12 (0.01)	0.10 (0.01)	0.303	0.147	–	–	–
Gait velocity CV (%)	0		5.93 (3.05)	4.76 (3.71)		0.612			0.209
	1	3.93 (4.24)	6.15 (1.68)	5.55 (2.20)	0.002	0.195	0.006	0.301	0.540
	2	5.90 (5.68)	6.25 (0.81)	6.90 (0.30)	0.443	<0.001	–	–	–
Swing time CV (%)	0		4.28 (0.59)	3.35 (3.13)		0.261			0.243
	1	3.25 (3.98)	4.30 (1.41)	3.83 (4.28)	0.022	0.104	0.028	0.302	0.911
	2	2.93 (2.95)	4.04 (1.35)	5.29 (1.32)	0.095	0.269	–	–	–

Median [IQR] values are reported for Improvers, Stables, and Deteriorators. Statistical analyses include ANOVA/Kruskal–Wallis tests with effect sizes eta squared ($\eta^{2}$) and post hoc *p*-values (Tukey’s HSD/Dunn’s test). Pairwise *p*-values were corrected using the Benjamini–Hochberg method. Statistical significance was defined as *p*-value < 0.05. (–): Not performed; post hoc tests were not conducted when the overall test was not significant. $\eta^{2}$ values were interpreted according to Cohen’s guidelines as small (∼0.01), medium (∼0.06), and large (≥0.14).

## Data Availability

The code used for machine-learning model development, spider plot generation, and the best-performing model is available on GitLab at https://gitlab.com/uniluxembourg/lcsb/dmg/francesca-boschi-phd-project/risk-of-gait-deterioration. Additional resources are available from the corresponding author upon reasonable request.

## Abbreviations

The following abbreviations are used in this manuscript:

AUC	Area Under the Curve
CV	Coefficient of Variation
H&Y	Hoehn and Yahr
IC	Initial Contact
IMU	Inertial Measurement Unit
LEDD	Levodopa Equivalent Daily Dose
ML	Machine Learning
PD	Parkinson’s Disease
PIGD	Postural Instability and Gait Difficulty
PwP	People with Parkinson’s Disease
RF	Random Forest
TC	Terminal Contact
UPDRS	Unified Parkinson’s Disease Rating Scale
UPDRSIII	Unified Parkinson’s Disease Rating Scale Section III

## Appendix A

Performances of multiple ML configurations are showed below. Separate models were trained using only clinical scores, only clinical scores with the PIGD score replacing the UPDRS III gait item, only sensor-derived gait parameters, and a combination of both clinical and sensor derived parameters, with and without substituting the UPDRS III gait item with the PIGD score.

### Appendix A.1. Best ML Model with Only Clinical Parameters (Without Substituting UPDRSIII Gait Item with PIGD Score)

**Best model setup**

- Number of features: 10
- Number of trees: 150
- Leaf size: 1

**Selected features**

Selected Feature	Average Importance	Standard Deviation
UPDRSIII gait	0.18	0.01
Age at study	0.17	0.00
Delta days	0.15	0.01
UPDRSIII total score	0.14	0.01
Disease duration	0.12	0.01
H&Y	0.09	0.00
UPDRSIII bradykinesia	0.06	0.00
UPDRSIII postural stability	0.04	0.00
Gender	0.03	0.00
UPDRSIII arise from chair	0.03	0.00

**Performance metrics**

AUC (±STD)	Accuracy % (±STD)
0.78 ± 0.01	60.72 ± 3.07

**Cumulative confusion matrix**

	Predicted labels		
True labels	Improvers	Stables	Deteriorators
Improvers	330	56	24
Stables	116	156	78
Deteriorators	68	94	188

### Appendix A.2. Best ML Model with Only Sensor-Derived Gait Parameters

**Best model setup**

- Number of features: 8
- Number of trees: 200
- Leaf size: 1

**Selected features**

Selected Feature	Average Importance	Standard Deviation
TC angle (°)	0.15	0.01
Swing time CV	0.15	0.01
Gait velocity CV	0.14	0.01
Stance time CV	0.15	0.01
Stride length (m)	0.15	0.01
Max lateral excursion (m)	0.13	0.00
Gait velocity (m/s)	0.13	0.01

**Performance metrics**

AUC (±STD)	Accuracy % (±STD)
0.62 ± 0.01	44.59 ± 1.86

**Cumulative confusion matrix**

	Predicted labels		
True labels	Improvers	Stables	Deteriorators
Improvers	220	96	94
Stables	71	174	105
Deteriorators	162	87	101

### Appendix A.3. Best ML Model with a Combination of Clinical and Sensor-Derived Gait Parameters (Without Substituting UPDRSIII Gait Item with PIGD Score)

**Best model setup**

- Number of features: 8
- Number of trees: 200
- Leaf size: 1

**Selected features**

Selected Feature	Average Importance	Standard Deviation
UPDRSIII gait	0.17	0.01
TC angle (°)	0.14	0.01
Stride length	0.13	0.01
Gait velocity (m/s)	0.13	0.01
Swing time CV	0.12	0.01
Stride length CV	0.14	0.01
Stance time CV	0.12	0.01
UPDRSIII bradykinesia	0.05	0.00

**Performance metrics**

AUC (±STD)	Accuracy % (±STD)
0.82 ± 0.01	67.21 ± 1.92

**Cumulative confusion matrix**

	Predicted labels		
True labels	Improvers	Stables	Deteriorators
Improvers	304	84	22
Stables	94	182	74
Deteriorators	34	56	260

### Appendix A.4. Best ML Model with Only Clinical Parameters (UPDRSIII Gait Item Substituted with PIGD Score)

**Best model setup**

- Number of features: 6
- Number of trees: 100
- Leaf size: 2

**Selected features**

Selected Feature	Average Importance	Standard Deviation
UPDRSIII total score	0.31	0.01
PIGD score	0.24	0.01
H&Y	0.17	0.01
UPDRSIII bradykinesia	0.13	0.01
UPDRSIII postural stability	0.08	0.01
UPDRSIII arise from chair	0.06	0.01

**Performance metrics**

AUC (±STD)	Accuracy % (±STD)
0.67 ± 0.01	50.54 ± 2.01

**Best model setup**

- Number of features: 10
- Number of trees: 200
- Leaf size: 1

### Appendix A.5. Best ML Model with a Combination of Clinical and Sensor-Derived Gait Parameters (UPDRSIII Gait Item Substituted with PIGD Score)

**Selected features**

Selected Feature	Average Importance	Standard Deviation
TC angle (°)	0.12	0.01
Stride length (m)	0.11	0.01
Max. lateral excursion (m)	0.11	0.01
Gait velocity (m/s)	0.11	0.01
Swing time CV	0.11	0.01
Stride length CV	0.10	0.01
Stance time CV	0.09	0.01
PIGD score	0.09	0.01
Gait velocity CV	0.09	0.01
UPDRSIII bradykinesia	0.07	0.01

**Performance metrics**

AUC (±STD)	Accuracy % (±STD)
0.71 ± 0.02	53.60 ± 2.05

**Cumulative confusion matrix**

	Predicted labels		
True labels	Improvers	Stables	Deteriorators
Improvers	253	89	68
Stables	64	188	98
Deteriorators	95	101	154

## References

[1] Brozova H., Stochl J., Roth J., Ruzicka E. (2009). Fear of falling has greater influence than other aspects of gait disorders on quality of life in patients with Parkinson’s disease. Neuro Endocrinol. Lett..

[2] Mirelman A., Bonato P., Camicioli R., Ellis T.D., Giladi N., Hamilton J.L., Hass C.J., Hausdorff J.M., Pelosin E., Almeida Q.J. (2019). Gait impairments in Parkinson’s disease. Lancet Neurol..

[3] Yang Y., Wang G., Zhang S., Wang H., Zhou W., Ren F., Liang H., Wu D., Ji X., Hashimoto M. (2022). Efficacy and evaluation of therapeutic exercises on adults with Parkinson’s disease: A systematic review and network meta-analysis. BMC Geriatr..

[4] Templeton J.M., Poellabauer C., Schneider S. (2022). Towards Symptom-Specific Intervention Recommendation Systems. J. Park. Dis..

[5] Nonnekes J., Nieuwboer A. (2018). Towards Personalized Rehabilitation for Gait Impairments in Parkinson’s Disease. J. Park. Dis..

[6] Goetz C.G., Fahn S., Martinez-Martin P., Poewe W., Sampaio C., Stebbins G.T., Stern M.B., Tilley B.C., Dodel R., Dubois B. (2007). Movement Disorder Society-sponsored revision of the Unified Parkinson’s Disease Rating Scale (MDS-UPDRS): Process, format, and clinimetric testing plan. Mov. Disord..

[7] Regnault A., Boroojerdi B., Meunier J., Bani M., Morel T., Cano S. (2019). Does the MDS-UPDRS provide the precision to assess progression in early Parkinson’s disease? Learnings from the Parkinson’s progression marker initiative cohort. J. Neurol..

[8] Hendricks R.M., Khasawneh M.T. (2021). An Investigation into the Use and Meaning of Parkinson’s Disease Clinical Scale Scores. Park. Dis..

[9] Hill E.J., Mangleburg C.G., Alfradique-Dunham I., Ripperger B., Stillwell A., Saade H., Rao S., Fagbongbe O., von Coelln R., Tarakad A. (2021). Quantitative mobility measures complement the MDS-UPDRS for characterization of Parkinson’s disease heterogeneity. Park. Relat. Disord..

[10] Schlachetzki J.C.M., Barth J., Marxreiter F., Gossler J., Kohl Z., Reinfelder S., Gassner H., Aminian K., Eskofier B.M., Winkler J. (2017). Wearable sensors objectively measure gait parameters in Parkinson’s disease. PLoS ONE.

[11] Hähnel T., Raschka T., Sapienza S., Klucken J., Glaab E., Corvol J.C., Falkenburger B.H., Fröhlich H. (2024). Progression subtypes in Parkinson’s disease identified by a data-driven multi cohort analysis. NPJ Park. Dis..

[12] Sotirakis C., Su Z., Brzezicki M.A., Conway N., Tarassenko L., FitzGerald J.J., Antoniades C.A. (2023). Identification of motor progression in Parkinson’s disease using wearable sensors and machine learning. NPJ Park. Dis..

[13] Espay A.J., Bonato P., Nahab F.B., Maetzler W., Dean J.M., Klucken J., Eskofier B.M., Merola A., Horak F., Lang A.E. (2016). Technology in Parkinson’s disease: Challenges and opportunities. Mov. Disord..

[14] Sapienza S., Tsurkalenko O., Giraitis M., Mejia A.C., Zelimkhanov G., Schwaninger I., Klucken J. (2024). Assessing the clinical utility of inertial sensors for home monitoring in Parkinson’s disease: A comprehensive review. NPJ Park. Dis..

[15] Skidmore F.M., Monroe W.S., Hurt C.P., Nicholas A.P., Gerstenecker A., Anthony T., Jololian L., Cutter G., Bashir A., Denny T. (2022). The emerging postural instability phenotype in idiopathic Parkinson disease. NPJ Park. Dis..

[16] Stebbins G.T., Goetz C.G., Burn D.J., Jankovic J., Khoo T.K., Tilley B.C. (2013). How to identify tremor dominant and postural instability/gait difficulty groups with the movement disorder society unified Parkinson’s disease rating scale: Comparison with the unified Parkinson’s disease rating scale. Mov. Disord..

[17] Ullrich M., Kuderle A., Hannink J., Del Din S., Gassner H., Marxreiter F., Klucken J., Eskofier B.M., Kluge F. (2020). Detection of Gait From Continuous Inertial Sensor Data Using Harmonic Frequencies. IEEE J. Biomed. Health Inform..

[18] Küderle A., Ullrich M., Roth N., Ollenschläger M., Ibrahim A.A., Moradi H., Richer R., Seifer A.K., Zürl M., Sîmpetru R.C. (2024). Gaitmap—An Open Ecosystem for IMU-Based Human Gait Analysis and Algorithm Benchmarking. IEEE Open J. Eng. Med. Biol..

[19] Goetz C.G., Stebbins G.T. (2004). Assuring interrater reliability for the UPDRS motor section: Utility of the UPDRS teaching tape. Mov. Disord..

[20] Hass C.J., Bishop M., Moscovich M., Stegemöller E.L., Skinner J., Malaty I.A., Wagle Shukla A., McFarland N., Okun M.S. (2014). Defining the Clinically Meaningful Difference in Gait Speed in Persons With Parkinson Disease. J. Neurol. Phys. Ther..

[21] Benjamini Y., Hochberg Y. (1995). Controlling the False Discovery Rate: A Practical and Powerful Approach to Multiple Testing. J. R. Stat. Soc. Ser. B (Methodol.).

[22] Ricciardi C., Amboni M., De Santis C., Ricciardelli G., Improta G., Iuppariello L., D’Addio G., Barone P., Cesarelli M. (2020). Classifying Different Stages of Parkinson’s Disease Through Random Forests. Mediterranean Conference on Medical and Biological Engineering and Computing.

[23] Ferreira M.I.A.S.N., Barbieri F.A., Moreno V.C., Penedo T., Tavares J.M.R.S. (2022). Machine learning models for Parkinson’s disease detection and stage classification based on spatial-temporal gait parameters. Gait Posture.

[24] Leal D.A.B., Dias C.M.V., Ramos R.P., Brys I. (2023). Prediction of dyskinesia in Parkinson’s disease patients using machine learning algorithms. Sci. Rep..

[25] Hu J., Szymczak S. (2023). A review on longitudinal data analysis with random forest. Brief. Bioinform..

[26] Probst P., Wright M.N., Boulesteix A.L. (2019). Hyperparameters and tuning strategies for random forest. Wiley Interdiscip. Rev. Data Min. Knowl. Discov..

[27] Urbanowicz R.J., Meeker M., La Cava W., Olson R.S., Moore J.H. (2018). Relief-based feature selection: Introduction and review. J. Biomed. Inform..

[28] Almgren H., Camacho M., Hanganu A., Kibreab M., Camicioli R., Ismail Z., Forkert N.D., Monchi O. (2023). Machine learning-based prediction of longitudinal cognitive decline in early Parkinson’s disease using multimodal features. Sci. Rep..

[29] Godi M., Arcolin I., Giardini M., Corna S., Schieppati M. (2021). A pathophysiological model of gait captures the details of the impairment of pace/rhythm, variability and asymmetry in Parkinsonian patients at distinct stages of the disease. Sci. Rep..

[30] Atrsaei A., Corrà M.F., Dadashi F., Vila-Chã N., Maia L., Mariani B., Maetzler W., Aminian K. (2021). Gait speed in clinical and daily living assessments in Parkinson’s disease patients: Performance versus capacity. NPJ Park. Dis..

[31] Welzel J., Wendtland D., Warmerdam E., Romijnders R., Elshehabi M., Geritz J., Berg D., Hansen C., Maetzler W. (2021). Step Length Is a Promising Progression Marker in Parkinson’s Disease. Sensors.

[32] Wang J., Gong D., Luo H., Zhang W., Zhang L., Zhang H., Zhou J., Wang S. (2020). Measurement of Step Angle for Quantifying the Gait Impairment of Parkinson’s Disease by Wearable Sensors: Controlled Study. JMIR mHealth uHealth.

[33] Baudendistel S.T., Haussler A.M., Rawson K.S., Earhart G.M. (2024). Minimal clinically important differences of spatiotemporal gait variables in Parkinson disease. Gait Posture.

[34] Costa T.M., Simieli L., Bersotti F.M., Mochizuki L., Barbieri F.A., Coelho D.B. (2022). Gait and posture are correlated domains in Parkinson’s disease. Neurosci. Lett..

[35] Wilson J., Alcock L., Yarnall A.J., Lord S., Lawson R.A., Morris R., Taylor J.P., Burn D.J., Rochester L., Galna B. (2020). Gait Progression Over 6 Years in Parkinson’s Disease: Effects of Age, Medication, and Pathology. Front. Aging Neurosci..

[36] Gaßner H., Trutt E., Seifferth S., Friedrich J., Zucker D., Salhani Z., Adler W., Winkler J., Jost W.H. (2022). Treadmill training and physiotherapy similarly improve dual task gait performance: A randomized-controlled trial in Parkinson’s disease. J. Neural Transm..

[37] Bowman T., Pergolini A., Carrozza M.C., Lencioni T., Marzegan A., Meloni M., Vitiello N., Crea S., Cattaneo D. (2024). Wearable biofeedback device to assess gait features and improve gait pattern in people with parkinson’s disease: A case series. J. Neuroeng. Rehabil..

[38] Scherbaum R., Moewius A., Oppermann J., Geritz J., Hansen C., Gold R., Maetzler W., Tönges L. (2022). Parkinson’s disease multimodal complex treatment improves gait performance: An exploratory wearable digital device-supported study. J. Neurol..

[39] Holden S.K., Finseth T., Sillau S.H., Berman B.D. (2017). Progression of MDS-UPDRS Scores Over Five Years in De Novo Parkinson Disease from the Parkinson’s Progression Markers Initiative Cohort. Mov. Disord. Clin. Pract..

[40] Chahine L.M., Siderowf A., Barnes J., Seedorff N., Caspell-Garcia C., Simuni T., Coffey C.S., Galasko D., Mollenhauer B., Arnedo V. (2019). Predicting Progression in Parkinson’s Disease Using Baseline and 1-Year Change Measures. J. Park. Dis..

[41] El Hayek M., Lobo Jofili Lopes J.L.M., LeLaurin J.H., Gregory M.E., Abi Nehme A.M., McCall-Junkin P., Au K.L.K., Okun M.S., Salloum R.G. (2023). Type, Timing, Frequency, and Durability of Outcome of Physical Therapy for Parkinson Disease: A Systematic Review and Meta-Analysis. JAMA Netw. Open.

[42] Packer E., Debelle H., Bailey H.G.B., Rehman R.Z.U., Yarnall A.J., Rochester L., Alcock L., Del Din S. (2025). Systematic review of wearables assessing medication effect on motor function and symptoms in Parkinson’s disease. NPJ Park. Dis..

[43] Kim S.M., Kim D.H., Yang Y., Ha S.W., Han J.H. (2018). Gait Patterns in Parkinson’s Disease with or without Cognitive Impairment. Dement. Neurocogn. Disord..

[44] Valldeoriola F., Coronell C., Pont C., Buongiorno M.T., Cámara A., Gaig C., Compta Y. (2010). Socio-demographic and clinical factors influencing the adherence to treatment in Parkinson’s disease: The ADHESON study: Socio-demographic and clinical factors in treatment of Parkinson’s disease. Eur. J. Neurol..

[45] Perepezko K., Hinkle J.T., Shepard M.D., Fischer N., Broen M.P., Leentjens A.F., Gallo J.J., Pontone G.M. (2019). Social role functioning in Parkinson’s disease: A mixed-methods systematic review. Int. J. Geriatr. Psychiatry.

